# EcoTILLING in *Beta vulgaris* reveals polymorphisms in the *FLC*-like gene *BvFL1* that are associated with annuality and winter hardiness

**DOI:** 10.1186/1471-2229-13-52

**Published:** 2013-03-25

**Authors:** Sebastian LM Frerichmann, Martin Kirchhoff, Andreas E Müller, Axel J Scheidig, Christian Jung, Friedrich J Kopisch-Obuch

**Affiliations:** 1 Plant Breeding Institute, Christian-Albrechts-University of Kiel, Olshausenstr. 40, Kiel, 24098, Germany; 2 Nordsaat Saatzucht GmbH, Böhnshauser Straße, Langenstein, 38895, Germany; 3 Strube Research GmbH & Co. KG, Hauptstr. 1, Söllingen, 38387, Germany; 4 Zoological Institute, Department of Structural Biology, Christian-Albrechts-University of Kiel, Am Botanischen Garten 1-9, Kiel, 24118, Germany

**Keywords:** *Beta vulgaris*, EcoTILLING, Diversity, Flowering, Winter hardiness, *BvFL1*

## Abstract

**Background:**

Sugar beet (*Beta vulgaris* ssp. *vulgaris* L.) is an important crop for sugar and biomass production in temperate climate regions. Currently sugar beets are sown in spring and harvested in autumn. Autumn-sown sugar beets that are grown for a full year have been regarded as a cropping system to increase the productivity of sugar beet cultivation. However, for the development of these “winter beets” sufficient winter hardiness and a system for bolting control is needed. Both require a thorough understanding of the underlying genetics and its natural variation.

**Results:**

We screened a diversity panel of 268 *B. vulgaris* accessions for three flowering time genes via EcoTILLING. This panel had been tested in the field for bolting behaviour and winter hardiness. EcoTILLING identified 20 silent SNPs and one non-synonymous SNP within the genes *BTC1*, *BvFL1* and *BvFT1*, resulting in 55 haplotypes. Further, we detected associations of nucleotide polymorphisms in *BvFL1* with bolting before winter as well as winter hardiness.

**Conclusions:**

These data provide the first genetic indication for the function of the *FLC* homolog *BvFL1* in beet. Further, it demonstrates for the first time that EcoTILLING is a powerful method for exploring genetic diversity and allele mining in *B. vulgaris*.

## Background

EcoTILLING is a fast and easy method to detect rare SNPs or small indels in target genes in natural populations. It is an adaptation of the TILLING (Targeting Induced Local Lesion In Genomes) technique that is used to detect point mutations in mutant populations [1]. In EcoTILLING, endonucleases such as CEL I are used to cut mismatched sites in the heteroduplex DNA formed by hybridization of different genotypes in a test panel. It is a cost effective technology as sequencing is limited to individual genotypes each representing a different haplotype. EcoTILLING has been used for the characterization of the genetic variability in *Arabidopsis thaliana* (thale cress) [2], *Musa* spp. (various banana species) [3], *Populus trichocarpa* (black cottonwood) [4], *Phaseolus vulgaris* (common bean) [5], and *Vigna radiata* (mung bean) [6]. Furthermore, it has been used for candidate gene-based detection of new alleles conferring resistance to biotic and abiotic stress in *Hordeum vulgare* (barley) [7], *Oryza sativa* (rice) [8,9], *Solanum tuberosum* (potato) [10]. *Cucumis* spp. (including cucumber) [11] and *Solanum lycopersicum* (tomato) [12]. EcoTILLING has not been reported for sugar beet (*Beta vulgaris* ssp. *vulgaris* L.) which contributes to 22% of the world production of white sugar [13].

Sugar beets are herbaceous, dicotyledonous plants that belong to the Amaranthaceae family (formerly Chenopodiaceae). The genus Beta is divided into the two sections *Corollinae* and *Beta*, the latter of which is further divided into cultivated beets (*B*. *vulgaris* ssp. *vulgaris*), wild sea beets (*B*. *vulgaris* ssp. *maritima* L.) and wild beets (*B*. *vulgaris* ssp. *adanensis*) [14]. Within *B*. *vulgaris* ssp. *vulgaris*, four cultivated groups can be distinguished: fodder beet, leaf beet, garden beet and sugar beet. While leaf beets and garden beets show an annual or biennial life cycle, sugar beets and also fodder beets are biennial plants that stay in the vegetative phase in their first year, forming a storage root with a high sucrose concentration of up to 20%. Both vernalization and long days are required for stem elongation (bolting) and flowering to occur in the second year of growth. Vernalization in sugar beet is achieved by exposure to cold temperatures for ten to 14 weeks.

Currently, sugar beets are cultivated as a spring sown crop in cool temperate climate regions. Seeds are sown in April and the roots are harvested starting in September. The late formation of a closed leaf canopy in spring is regarded as the main factor limiting beet yield [15]. One strategy to overcome this is the production of autumn sown winter beets which develop a closed canopy earlier in spring. However, breeding of autumn sown winter beets requires sufficient winter hardiness to survive the winter and a system for bolting control which allows bolting for seed production but represses bolting after winter during crop production [16]. With key regulators of flowering and bolting in *B*. *vulgaris* recently having been identified [17,18], bolting control may be achieved by genetic modification which on the one hand allows suppression of bolting after winter for cultivation of beets, but on the other hand enables bolting for seed production [16].

In order to avoid an untimely transition to the extremely cold-sensitive generative phase [19] before or during winter, and to facilitate the accumulation of sufficient resources for reproduction, winter-annual and biennial plants growing in temperate zones require vernalization for induction of flowering. Cultivated beets are biennials, whereas annual beets without a requirement for vernalization are frequently observed in wild beet populations [20,21]. The vernalization response in biennial beets is mediated by the *FLOWERING LOCUS T* (*FT*) homolog *BvFT1*, which in contrast to the promotive action of *FT* in Arabidopsis functions as a repressor of flowering [17]. Similar to *FLC*, *BvFT1* is gradually down-regulated during the prolonged cold of winter [17]. In annual beets, *BvFT1* is not expressed even in the absence of vernalization and was shown to be negatively regulated by the pseudo-response regulator gene *BOLTING TIME CONTROL 1* (*BTC1*), formerly referred to as *BvBTC1*[18]. This gene is located at the bolting locus *B* and is a major determinant of the annual growth habit in beet. The dominant *BTC1* allele promotes bolting in annuals in response to long days, whereas biennials carry a partial-loss-of-function allele which is not able to mediate the promotive effect of long days without prior vernalization [18]. All cultivated (biennial) beet accessions tested were found to carry the same haplotype whereas the vast majority of wild sea beets harbour haplotypes which resemble the *BTC1* allele found in annual reference accessions [18].

Several other genes in beet have been identified on the basis of homology to floral transition genes in Arabidopsis, including the central regulator of vernalization requirement and response in this species, *FLOWERING LOCUS C* (*FLC*) [22,23]. The *FLC-LIKE 1* gene *BvFL1* is gradually down-regulated during a prolonged exposure to cold under continuous light [24]. Constitutive expression of *BvFL1* in an *FLC* null mutant of Arabidopsis significantly delayed flowering, suggesting at least partial evolutionary conservation of function between *FLC* homologs in Arabidopsis and beet.

Interestingly, flowering time control genes also seem to affect frost tolerance, which is the most important factor contributing to winter hardiness [25-27]. Plants can further increase their frost tolerance by a gradual adaptation of the metabolism during a hardening process that occurs at non-freezing temperatures below 10°C. In Arabidopsis, frost tolerance is regulated by the C-REPEAT BINDING FACTOR (CBF) transcription factor family, with plants constitutively overexpressing *CBF* genes showing an increase in frost tolerance [28] and elevated levels of *FLC* expression [29]. Deng et al. [30] reported that *FLC* plays a dual role in flowering time control and cold stress response. Interestingly, a recent study suggested that the recruitment of a repressive chromatin complex at the *FLC* locus involves the cold-induced expression of a long non-coding RNA, termed *COLDAIR*, from intron 1 of *FLC*[31].

In the present study, we established EcoTILLING in *B*. *vulgaris* to survey a large panel of cultivated and wild beets for allelic variants of candidate genes for regulators of vernalization requirement and/or winter hardiness. This panel had been phenotyped before for variation in the occurrence of bolting before winter (i.e. in the absence of vernalization) and survival rates after winter [32]. As candidate genes we chose (i) *BTC1* and (ii) *BvFT1*, because of their known functions in the regulation of vernalization requirement and response in beet, and (iii) *BvFL1*, because of the regulatory role of its homolog *FLC* in both vernalization and cold stress response in Arabidopsis. We found that haplotype variation at the *BvFL1* locus was associated with variation in bolting rate before winter and survival rate after winter. These data provide the first genetic indication for the function of the *FLC* homolog *BvFL1* in beet, and are relevant for sugar beet breeding and our understanding of the bolting time control network in Beta.

## Results

### Phenotyping and model-based analyses of population structure

**Figure 1:**
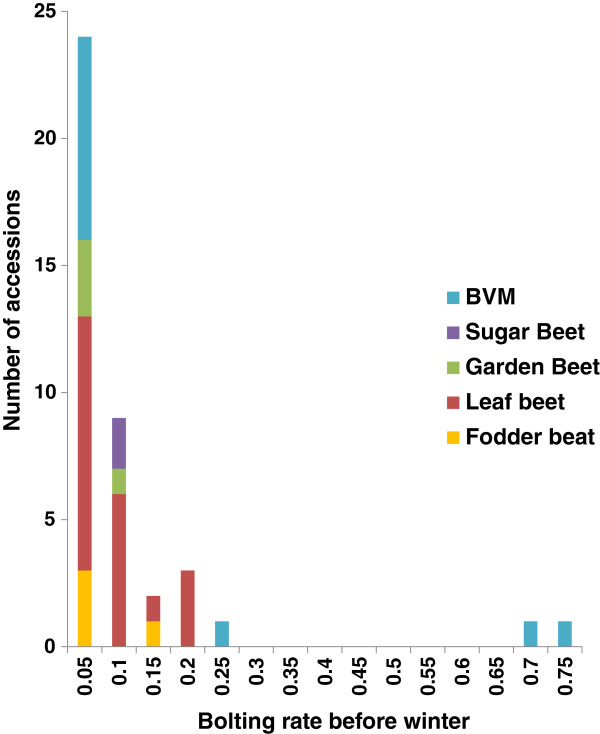
**Distribution of bolting rate before winter.** Distribution of bolting rate before winter among a subset of 41 *B*. *vulgaris* accessions tested in four different environments. BVM = *B*. *vulgaris* ssp. *maritima*.

**Figure 2:**
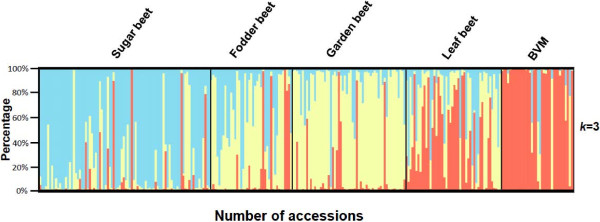
**Overview of population structure analysis.** Population structure of 268 *B*. *vulgaris* accessions based on 40 AFLP markers under the assumption of *k* = 3 subpopulations. Each *B*. *vulgaris* accession is represented by one bar that is divided in up to k segments, each proportional to the inferred subpopulation. Accessions are grouped by their respective *B*. *vulgaris* group. BVM = *B*. *vulgaris* ssp. *maritima*.

In fall 2009, 41 out of the 268 accessions sown in the field in July or August at four different locations, had at least one plant which had started bolting before the first frost. These accessions included two sugar beets (2.2% of accessions tested), four garden beets (6.9%), four fodder beets (10.0%), 20 leaf beets (42.3%) and eleven wild sea beets (*B. vulgaris* ssp. *maritima*) (31.4%). Across all four environments, bolting rates for the 41 accessions ranged from 0.05 to 0.75 (Figure 1). Variation for survival rate survival rate after winter across eight environments in 2008/09 and 2009/10 was described in detail by Kirchhoff et al. [32], and ranged from 0.07 to 0.66. On average, sugar beet accessions performed best (0.39) while fodder beet and garden beet performed worst (0.24 and 0.19, respectively). The largest variation for survival rate was found in *B. vulgaris ss*p*. maritima* followed by leaf beets, whereas sugar beets showed the smallest variation. Population structure was analysed by a model-based method using the genotypic data for 40 polymorphic AFLPs detected among the 268 accessions. An independent calculation of *k* was repeated six times for each value of *k* from *k* = 1 to *k* = 8. The log probability *L*(*K*) increased sharply from *k* = 1 to *k* = 3, but only slowly after *k* = 3 (Additional file 1). When *k* is approaching a true value, *L(K)* plateaus or continues to increase slightly [33]. Therefore the structure analysis suggested the presence of three subgroups (*k* = 3), where most of the sugar beets (0.81) fell in the first group, most of the fodder beets (0.61) and garden beets (0.86) in the second group, and most of the leaf beets and *B. vulgaris ss*p*. maritima* in the third group (Figure 2). To further increase confidence in the *k* value estimate, we calculated ΔK and obtained the highest ΔK value (60.04) for *k* = 3 (Additional file 2).

### Amplification of candidate genes

For the three candidate genes *BvFL1*, *BvFT1* and *BTC1* we designed 24 primer pairs, of which five primer pairs passed the primer “crash-test” and therefore were suitable for EcoTILLING. The genomic sequence of *BvFL1* [GenBank: DQ189214.1 and DQ189215.1] was taken from Reeves et al. [24] while the genomic sequence of *BvFT1* [GenBank: HM448909.1] and *BTC1* [GenBank: HQ709091.1] were taken from Pin et al. [17,18]. We adopted a primer pre-screen as described by Weil and Monde [34] to test for the occurrence of unwanted amplification from single primers prior to the costly synthesis of labelled primers. Amplification from the five successfully tested primer pairs resulted in a total amplicon length of 4,234 bp (Table 1). The remaining primer combinations were not suitable for EcoTILLING due to miss-priming and single primer amplification revealed in the primer crash-test (data not shown).

**Table 1:** Overview of candidate genes investigated with EcoTILLING

**Gene**	**Genomic size**	**Protein domain(s)**	**Amplicon size(s)**	**Primer name and sequence (5’-3’)**	**Length and portion of genomic sequence covered by EcoTILLING**	**Portion of reading frame covered by EcoTILLING**
*BvFL1*	8.62 kb	MADS box/K box	FL1a: 977 bp FL1b: 632 bp	FL1a-fw tcggactttccctataagct FL1a-rv cacgtgaatcgttacagaca FL1b-fw gctgatagtctgtcccttttgtc FL1b-rv tgactccaacaccacgatgca	1,609 bp (19%)	62%
*BvFT1*	7.53 kb	PEBP	FT1a: 916 bp FT1b: 713 bp	FT1a-fw tggtacgtgtatgaaacagaagctg FT1a-rv catcaactccatatttggggtg FT1b-fw acccatctatacttgtcgatgacc FT1b-rv caatggggaagtggttcacact	1,629 bp (22%)	92%
*BTC1*	11.27 kb	REC, CCT	BTC1: 996 bp	BTC1-fw cagctgtaggatgttatcgtgctgag BTC1-rv agtaggtgataaggacaagacattgc	992 bp (9%)	15%

The gene names, genomic sizes in kilo base pairs (kb), protein domains, sizes of amplicons in base pairs (bp) per gene, primer names and sequences, and the sizes of the amplicons in base pairs (bp) are given. Genomic size was defined here as the size of the genomic sequence of a gene from the start to the stop codon plus 1 kb each upstream and downstream. The genomic size given for *BvFL1* refers to the known portion of the sequence. A large part of intron 1 was not sequenced (Reeves et al. [24]) and is not considered here. Furthermore, the length of the investigated genomic sequence and the percentage of the open reading frame which was investigated via EcoTILLING are given.

**Figure 3:**
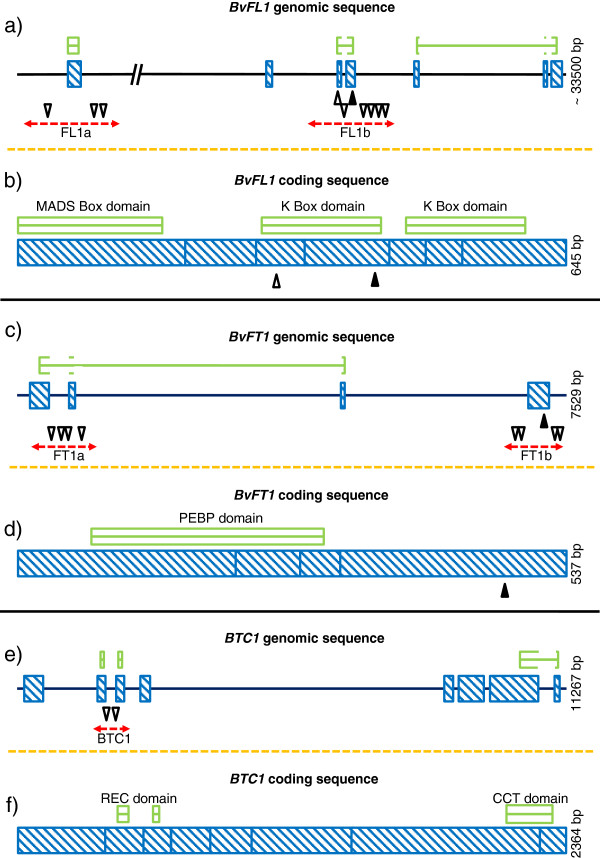
**Flowering time genes *BvFL1,BvFT1 *and *BTC1 *and distribution of polymorphisms.** Shown are graphical outputs from PARSESNP. **a**), **c**), **e**) Genomic sequences with intron/exon structure. **b**), **d**), **f**) Coding sequences. Red dotted arrowed lines indicate the amplicons investigated by EcoTILLING. Blue striped boxes indicate exons. White arrowheads that point downwards indicate changes in non-coding regions. Black arrowheads that point upwards indicate changes that induce missense mutations in the protein products. White arrowheads that point upwards indicate silent changes. Green boxes mark important protein domains.

For *BvFL1* we could amplify two regions (Figure 3). The first amplicon (‘FL1a’) covered 977 bp of the promoter, exon 1 (including the 5'-UTR and the 5' region of the coding sequence), and part of intron 1. The second amplicon (‘FL1b’) spanned a region of 632 bp in exons 3 and 4 and the intervening intron. These regions were chosen because they contain the promoter region, the region encoding the MADS box domain as well as a TGTGAT sequence motif (K box) which is associated with transcription factor binding activity. Thus, 62% of the *BvFL1* ORF was covered. Furthermore, we targeted the first intron, which is known to include a number of regulatory regions in *Arabidopsis*[35]. For *BvFT1* we amplified a 916 bp region (‘FT1a’) that extends from the 5'-UTR to the 5' region of the coding sequence in exon 1. A second amplicon (‘FT1b’) spanned 713 bp and was located in exon 4 and the 3'-UTR (Figure 3). Both regions together cover 92% of the ORF and were chosen because they contain parts of the promoter and the PEB domain (Table 1). In *BTC1* we amplified a 992 bp fragment (‘BTC1’) of the conserved response regulator receiver (REC) domain region (Figure 3), which covered 15% of the ORF (Table 1).

### Genetic diversity, SNP densities and haplotype frequencies

Across the five amplicons (FL1a, FL1b, FT1a, FT1b and BTC1) a total of 21 SNPs were identified among the 268 accessions tested by LI-COR analyses. Eighteen SNPs were located in introns, while the remaining three SNPs were located in exon 4 of *BvFT1* and in exon 3 and exon 4 of *BvFL1*, the latter SNP of which was non-synonymous. The number of polymorphisms varied from gene to gene and had an overall density of 5.3 SNP/kb. The lowest SNP density was found in *BTC1* (2.01 SNP/kb), whereas the highest SNP density occurred in FL1b (9.82 SNP/kb) (Additional file 3). The SNP allocations and gene structures are shown in Figure 3. To evaluate the efficiency of EcoTILLING in *B. vulgaris* we estimated the rate of false negatives by sequencing all amplicons in four selected accessions, which resulted in a false negative rate of 5%. Sequencing of FT1a, FT1b, FL1a and BTC1 did not reveal additional SNPs which had not been already identified by LI-COR analyses. For the amplicon FL1b one additional SNP was identified after sequencing in a single accession. This SNP was not detected for any of the 268 accession by EcoTILLING on the LI-COR.

**Figure 4:**
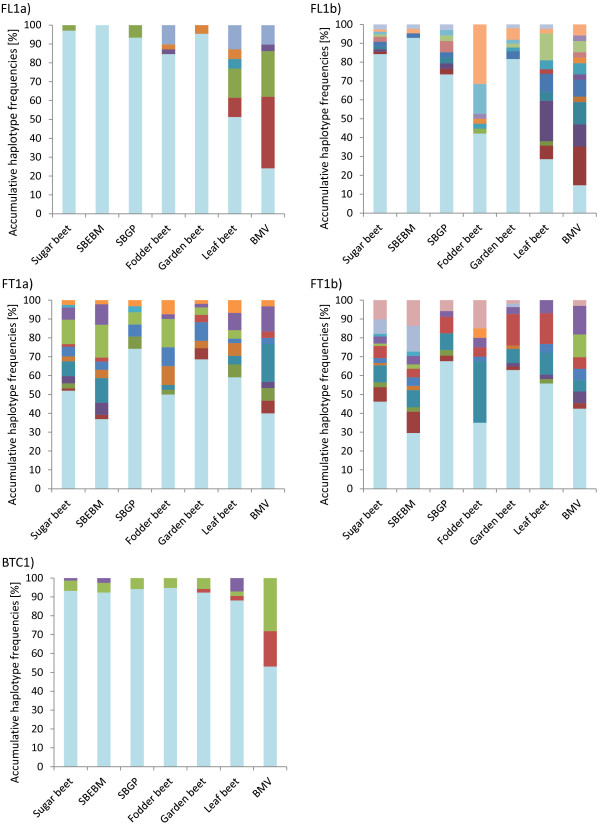
**Haplotype diversity diagram.** Shown is the haplotype distribution for the amplicons FL1a, FL1b, FT1a, FT1b and BTC1. The percentage of haplotypes for each amplicon is given for each *B. vulgaris* form. Haplotypes are colour coded. The reference haplotype H0 is always shown in light blue, while non-reference haplotypes are shown in various colours. The *B. vulgaris* form is given on the x-axis. SBEBM = Sugar beet elite breeding material, SBGP = Sugar beet germplasm, BVM = *B*. *vulgaris* ssp. *maritima*.

The mean non-reference nucleotide frequencies (NNFs; s. Materials and Methods) for *BvFL1* (0.18) and *BvFT1* (0.17) are similar across all accessions tested, but varied between individual *B. vulgaris* forms, ranging from 0.12 in garden beets to 0.23 in *B. vulgaris ss*p*. maritima* for *BvFT1*, and from 0.04 in garden beets to 0.55 in *B. vulgaris ss*p*. maritima* for *BvFL1* (Additional file 4). The mean NNF for *BTC1* over all *B. vulgaris* forms was 0.07, and ranged from 0.03 in fodder beets to 0.30 in *B. vulgaris ss*p*. maritima*. We identified 55 haplotypes across all five amplified candidate regions. The numbers of haplotypes ranged from four in amplicon BTC1 to 18 in amplicon FL1b. Sixty per cent of the detected haplotypes were rare, occurring at frequencies below 0.05. The reference haplotype (H0) was the most common in each amplicon, with frequencies ranging from 0.49 for FT1b_H0 to 0.87 for BTC1_H0 (Figure 4). The non-reference haplotype frequencies (NHF) ranged from 0.01 to 0.38 across the amplicons within the distinct *B. vulgaris* forms (Figure 4). The highest NHF was observed in *B. vulgaris ss*p*. maritima*, while garden and sugar beets had the lowest NHF. Gene diversity (Ht) for each amplicon and within each cultivar group is displayed in Additional file 5. Ht range was lowest in the amplicon FT1b (0.16 to 0.30) and highest in the amplicon FL1a (0.02 to 0.51). The highest and lowest diversity for the *B. vulgaris* forms was observed in amplicon FL1a for *B. vulgaris ss*p*. maritima* (0.51) and garden beets (0.02), respectively. Subdividing the 88 investigated sugar beet accessions into 49 accessions of elite breeding material (SBEBM) provided by Strube GmbH & Co. KG (Söllingen, Germany) and the remaining 39 accessions of sugar beet germplasm (SBGP, mostly composed of various gene bank accessions) revealed a trend towards lower diversity in the amplicons FL1a, FL1b and BTC1, while the diversity increased in FT1a and FT1b (Additional file 6).

### *BvFL1* sequence variations are associated with bolting and survival rate

**Figure 5:**
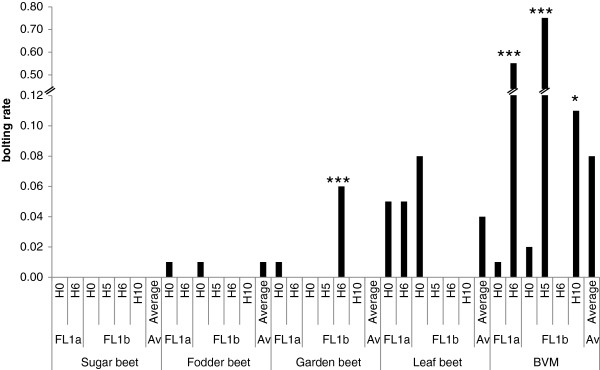
***BvFL1 *haplotype effect on bolting rate before winter.** Graphic representation of the effect of *BvFL1* haplotypes of the amplicons FL1a and FL1b on bolting rate before winter in five *B. vulgaris* forms. * or *** mark non-reference haplotypes with a significantly different bolting rate from reference haplotypes FL1a_H0 or FL1b_H0 (P < 0.05 or P < 0.0001, respectively); BVM = *B*. *vulgaris* ssp. *maritima*.

**Figure 6:**
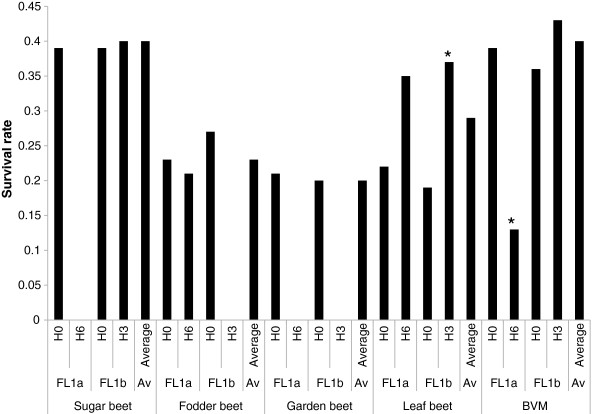
***BvFL1 *haplotype effect on survival rate after winter.** Graphic representation of the effect of *BvFL1* haplotypes of the amplicons FL1a and FL1b on survival rate after winter in five *B*. *vulgaris* forms. * marks non-reference haplotypes with a significantly different survival rate from the reference haplotypes FL1a_H0 or FL1b_H0 (alpha = 0.05); BVM = *B*. *vulgaris* ssp. *maritima*.

Based on the Q matrix for *k* = 3, associations with bolting rate, survival rate, and survival rate with bolting rate as cofactor were each significant (*P* ≤ 0.05) for the amplicon FL1a and for the amplicon FL1b (Table 2). Dunnet comparisons revealed that *B. vulgaris ss*p*. maritima* accessions with haplotype FL1a_H6, FL1b_H5 and FL1b_H10 had a significantly higher bolting rate of 55% (*P* < 0.0001), 75% (*P* < 0.0001) and 11% (P < 0.05), respectively, compared to 1% for FL1a_H0 and 2% for FL1b_H0 (Additional file 6). Furthermore, garden beet accessions with haplotype FL1b_H6 bolted before winter with a bolting rate of 6% (*P* < 0.0001) compared to 1% for accessions with the reference haplotype FL1b_H0 (Figure 5). Dunnet comparisons for survival rate revealed that *B. vulgaris ss*p*. maritima* accessions with haplotype FL1a_H6 had a significantly lower survival rate of 13% (*P* = 0.015) compared to 39% observed for accessions with the reference haplotype FL1a_H0 (Additional file 7). By contrast, leaf beet accessions with the haplotype FL1b_H3 had a significantly higher survival rate of 37% (*P* = 0.012) compared to 19% of accessions with the reference haplotype FL1b_H0 (Figure 6). DNA sequences of significant haplotypes of *BvFL1* are shown in Additional file 8.

**Table 2:** Significant associations of bolting and survival rate with amplicon

**Trait**	**Amplicon**	**F**^**d)**^	**P**^**e)**^	**R**^**2 f)**^
BR^a)^	FL1_a	9.04**	6.47E^-11^	0.22
BR	FL1_b	11.28**	2.65E^-23^	0.44
SR^b)^	FL1_a	7.17**	1.44E^-08^	0.13
SR	FL1_b	2.91**	7.72E^-05^	0.13
SR/BR^c)^	FL1_a	6.54**	9.52E^-08^	0.11
SR/BR	FL1_b	2.90**	1.14E^-04^	0.12

a) BR = bolting rate, b) SR = survival rate c) SR with BR as cofactor, d) F value from the F test on marker, e) Bonferroni corrected p value, f) R^2^ is the fraction of the total variation explained by the amplicon.

**(P < 0.001) indicates the amplicon is highly significantly associated with trait.

## Discussion

This is the first report of EcoTILLING applied to *B. vulgaris*. We established EcoTILLING based on a panel of 268 accessions representing the wild and domesticated gene pool of *B. vulgaris*. In this panel we successfully screened the allelic variation in three genes that are candidates for regulators of vernalization requirement and/or winter hardiness. As a result we were able to provide a snapshot of the species-wide diversity within these genes. Further, we identified haplotypes that are associated with bolting rate before winter and with survival rate, which in turn might be useful for improvement of winter hardiness in sugar beets. Our results show that EcoTILLING is a suitable and cost effective method for allele mining in *B. vulgaris*.

In most EcoTILLING protocols heteroduplexed DNA is digested by purified CEL I endonuclease. Instead of the purified enzyme, Till et al. [36] and Galeano et al. [5] used celery juice obtained from salted out or dialyzed crude celery extract for EcoTILLING screens of *Arabidopsis thaliana* and *Phaseolus vulgaris*, respectively. We went a step further and used the crude celery extract (CCE) without further processing, and observed the same activity as compared to the commercial CEL I enzyme Surveyor® (data not shown). Also, CCE was very stable and kept its activity for weeks even when stored at 4°C. As using CCE eliminates the need for special enzyme purification steps like chromatography and specialized laboratory equipment, this increases the cost efficiency of EcoTILLING. We were further able to show that once suitable primers are designed, EcoTILLING provides a high throughput method for the analysis of natural nucleotide diversity in *B*. *vulgaris*. Also, EcoTILLING is a rather cost effective method. When evaluating LI-COR gels, signals can be grouped according to size and pattern, and only a limited number of samples per group need to be sequenced to break down the detected variation to the nucleotide level. This drastically reduces the sequencing costs, in our case by 1/3. If only SNPs/haplotypes with effect on the phenotype of interest are sequenced, costs can be further reduced. However, it has to be considered that EcoTILLING is prone to false negative detection because some fragment sizes are masked by background “noise”, due to miss-priming, or because of weaker fluorescence toward the top of each lane and increasing fluorescence “noise” toward the bottom [3,37,38]. The false negative rate in our case was 5% which is similar to rates reported in human [39] and banana [3] EcoTILLING.

The population structure analysis by AFLP markers indicates that the *B. vulgaris* accessions can be grouped into three groups (*k* = 3). Under consideration of the phenotypic classification of the panel (gene bank information as well as our field observations on plant habitus), the three groups can be referred to as a sugar beet group, a fodder beet and garden beet group, and a group comprising leaf beets and wild sea beets. This reflects the evolutionary history of Beta and the selection intensity during the past 200 years of beet breeding. A similar structure has also been described by Jung et al. [40] and by McGrath et al. [41] after genotyping with completely different marker systems. Both groups report that sugar beets can be clearly distinguished from *B. vulgaris* ssp. *maritima*. In our study, a few accessions were classified differently by genotype (according to AFLP analysis) than by phenotype (see Figure 2). Regarding *B.vulgaris ss*p. *maritima* this could hint at gene flow from cultivated forms into wild material, either in their natural habitat or during propagation by gene banks. At the same time, classification by phenotype was sometimes ambiguous. For instance ‘Patak’ accessions from India (PI 116809 and PI 121838), although cultivated, showed a plant habitus whose classification into *B.vulgaris ss*p. *maritima* seems more reasonable than classification into any of the cultivated forms. Interestingly, both approaches to account for population structure resulted in significant associations of the same amplicons, hinting at the robustness of the results. Nevertheless, to account for genotyping errors as a source for putative misclassification by STRUCTURE, genotypic outliers were removed from the dataset for a further analysis by TASSEL. These outliers were sugar beet, fodder beet and table beet accessions with an estimated portion of non-cultivated beet genome > 50% and *B.vulgaris ss*p*. maritima* accessions with an estimated portion of cultivated beet genome > 50% (see Figure 2). In this analysis previous associations were still highly significant (data not shown).

Comparing all five *B. vulgaris* forms we observed the highest genetic diversity for the investigated genes in *B. vulgaris ss*p*. maritima*. This is indicated by a mean NNF of 0.36 compared to 0.19 in leaf beets followed by fodder beets (0.12), sugar beets (0.08) and garden beets (0.07). The same trend is observed when looking at the average NHF and Ht. Our findings are in accordance with Jung et al. [40] and Fénart et al. [42] who reported a higher genetic diversity in *B. vulgaris ss*p*. maritima* compared to sugar beets. As selection results in a loss of genetic diversity, it is not surprising that the genetic diversity in *B. vulgaris ss*p*. maritima* appears to be higher not only compared to sugar beet but also in comparison to all four cultivar groups taken together. Crop evolution is best understood for sugar beet which has been affected by founder effects as it was derived from a single fodder beet population and also by genetic bottlenecks through introgression of a series of traits from a limited number of genetic resources [43-45]. This explains why sugar beet together with red table beet showed the lowest diversity.

In our study the genetic diversity in sugar beet based on Ht ranged from 0.03 to 0.28 for the single amplicons with an average of 0.17. These estimates are likely to be upward biased as we could not distinguish between the occurrence of non-reference nucleotides in the heterozygous or homozygous state. Nevertheless, gene diversity in our study is lower compared to Li et al. [46] and McGrath et al. [41]. However, in contrast to Li et al. and McGrath et al. we estimated the genetic diversity for nucleotide polymorphisms in three genes that may have been under selective pressure. This is especially the case for *BvFL1* where we estimated Ht values of 0.03 and 0.12 for FL1a and FL1b, respectively, and also for *BTC1* (Ht = 0.06). This could be the effect of selection for bolting resistance to prevent bolting caused by late frosts after sowing in spring. Comparing sugar beet elite breeding material with sugar beet germplasm, the genetic diversity turned out to have been further decreased by selection for *BTC1* and *BvFL1* (see also Additional file 5). At the same time *BvFT1* showed even more diversity in SBEBM indicating that this gene is obviously not under selective pressure. This is somehow surprising, as *BvFT1* was shown to play a key role in bolting suppression under non-inductive conditions [17]. Still, these data have to be interpreted with care, as sample sizes are moderate and the SBEBM material represents only one breeding company.

For *BvFL1*, we were able to detect an association with bolting. Four haplotypes of this gene (FL1a_H6, FL1b_H5, FL1b_H6 and FL1b_H10) had a significant effect on bolting rate before winter in *B. vulgaris ss*p*. maritima* and/or garden beets. Although variation in *FLC* is known to affect flowering time in *A. thaliana*[47], the role of *FLC*-like genes outside the *Brassicaceae* is not well understood [16], and a functional analysis of *BvFL1* in *B. vulgaris*, e.g. through mutational or transgenic approaches, is still lacking. Effects on bolting rate were not observed for all *B. vulgaris* forms, which in part may be due to the absence of the divergent haplotypes that affect bolting rate in *B. vulgaris ss*p*. maritima* or garden beet. The complete absence of these haplotypes in sugar beet may reflect the breeding history of sugar beet, during which breeders strongly selected against bolting before vernalization [48,49].

As *BTC1* is known to be a major factor controlling bolting without prior vernalization in beets [18], we expected an effect of *BTC1* sequence variations on bolting before winter. However, this was not observed here. This may be due in part to an underrepresentation of annual BTC1 alleles in our panel or the fact that the current analysis was limited to a relatively small portion of the coding sequence of *BTC1* (15%; Table 1), and did not include the promoter. Although *BvFT1* is known to respond to vernalization and is down-regulated by cold temperatures, which in turn enables induction of flowering [17], we also did not detect an effect of haplotype variation in this gene on bolting rate. While other reasons for this cannot be excluded, as discussed for *BvFL1* and *BTC1*, it is also conceivable that possible phenotypic effects of haplotype variation at *BvFT1* are difficult to detect under the environmental conditions present in the current study.

Besides significant effects on bolting rate, plants with haplotypes FL1a_H6 and FL1b_H3 showed a significant impact on survival rate after winter in *B. vulgaris ss*p*. maritima* and leaf beets, respectively. A similar effect has been shown before for *A. thaliana* ecotypes, where a SNP in intron 1 of *FLC* led to a 1.6-fold increase in winter survival rates in genotypes carrying a functional *FRI* allele [50]. The authors suggested that survival after winter is associated with time to bolting. Similarly, we found that the survival rate of truly biennial (vernalization requiring) leaf beet accessions with the FL1b_H3 haplotype was higher (by 20%) when compared to the reference haplotype FL1_H0 (p = 0.006, data not shown). Hence, winter hardiness in sugar beet might be improved by introgressing FL1b_H3 from leaf beet. Interestingly, *B. vulgaris ss*p*. maritima* accessions with FL1a_H6, which had a lower survival rate than accessions with the reference haplotype, also showed an increased bolting rate. Furthermore, after removal of *B. vulgaris ssp. maritima* accessions which bolted before winter, the accessions with haplotype FL1a_H6 did not have a significant effect anymore. Therefore, the lower survival rate observed for this haplotype might be a direct physiological effect of bolting before winter as plants in the generative phase are more frost sensitive [19]. However, by using bolting rate as a cofactor in a further TASSEL analysis, we can exclude that increased frost sensitivity in the generative phase is the mere cause for association of *BvFL1* with survival rate since this association stayed significant. Interestingly, Seo et al. [29] reported that transient cold temperatures and overexpression of CBFs lead to elevated *FLC* expression and delayed flowering, suggesting a possible role of *FLC* in cold stress response in *A. thaliana* and, by analogy, a possible explanation for the detected effect of *BvFL1* haplotypes on survival rate in *B. vulgaris*. Effects on survival rate could not be observed consistently for both haplotypes throughout all *B. vulgaris* forms. This may in part be due to the absence of the two haplotypes in some of the other *B. vulgaris* forms (see Additional file 7). Similar to bolting rate, the absence of haplotype effects on survival rate in some *B. vulgaris* forms might also be due to the polygenic inheritance of survival rate.

Among the SNPs underlying haplotypes FL1a_H6, FL1b_H3, FL1b_H5, FL1b_H6, and FL1b_H10, two are located in an exon. The SNP in exon 3 is synonymous, whereas the SNP in exon 4 is non-synonymous, leading to an amino acid substitution from valine to isoleucine. The other SNPs identified in *BvFL1* are silent as they are located in introns, including intron 1. These SNPs might influence gene function by affecting the transcriptional regulation of *BvFL1*, as was reported for intronic polymorphisms in *FLC* in Arabidopsis [51,52]. Also, Heo and Sung [31] reported that the regulatory non-coding RNA *COLDAIR* is expressed from intron 1 of *FLC*. Finally, the increase in winter survival rates observed for an allelic variant of *FLC*[50] was also associated with polymorphisms in intron 1. As with association studies in general, it cannot be excluded that the functional polymorphisms for the traits investigated are located outside the amplified gene regions and that the SNPs detected here are merely linked to these polymorphisms.

## Conclusions

In conclusion, our study demonstrates that EcoTILLING can be successfully employed in *B. vulgaris* to survey a large panel of plant accessions for allelic variants in different candidate genes. Our data also provide the first genetic indication that an *FLC* homolog indeed may also affect flowering time (and winter survival) in a species which is only distantly related to *A. thaliana*. The above described panel of diverse *B. vulgaris* forms is an excellent resource to identify allelic variation in additional flowering time control genes such as *BvFT2* or candidate genes for agronomic traits such as stress response and plant architecture. Allelic variants identified by EcoTILLING can be used to introduce new genetic variation into elite beet breeding material.

## Methods

### Plant material and phenotypic data

Phenotypic data for bolting before winter and winter hardiness were taken from a recent study described in detail by Kirchhoff et al. [32]. In short, a panel of 396 *B. vulgaris* accessions covering a wide range of genetic diversity was tested for winter hardiness in a replicated overwintering field experiment in eight environments at five different locations in Germany and Belarus in the winters of 2008/09 and 2009/10. Survival rates were determined as the fraction of surviving individuals among all plants of a given accession ranging from 0 to 1, where 0 means no plants survived and 1 means all plants from one accessions survived. The mean survival rates were estimated as best linear unbiased predictors (BLUPs) for each accession across all environments. Accordingly, bolting rates before winter were determined in the 2009/10 environments as the fraction of bolting individuals among all plants of a given accession ranging from 0 to 1, where 0 means none of the plants bolted and 1 means all plants of a given accession bolted. Recording time was before the first frost (2 December 2009). To avoid unbalanced data, we reduced the data set to a subpanel of 268 accessions that were tested in all environments. These comprise the four cultivar groups fodder beet (40), leaf beet (47), garden beet (58) and sugar beet (88), as well as 35 *B. vulgaris ss*p*. maritima* accessions. The 88 sugar beets can be further subdivided into 49 elite accessions (sugar beet elite breeding material, SBEBM) provided by Strube GmbH & Co. KG (Söllingen, Germany) and 39 mostly gene bank accessions of various origins (sugar beet germplasm, SBGP).

### DNA isolation and screening for polymorphisms

DNA was isolated from freeze dried leaf samples taken from up to eight plants per accession. This was done with a NucleoSpin® 96 Plant II Kit (MACHEREY-NAGEL GmbH & Co. KG, Düren, Germany) as recommended by the manufacturer on a Tecan “Freedom Evo” Robot. DNA concentration was measured via the Tecan Robot using a photometer and SYBR® Green (Invitrogen GmbH, Darmstadt, Germany) and normalized with DNase free water to a final concentration of 10 ng/μl in a total volume of 160 μl. The 268 DNA samples representing the 268 *B.vulgaris* accessions of the test panel were each pooled 1:1 with DNA of the biennial sugar beet 93161P as reference type and stored in 96 well plates. 93161P is an inbred line homozygous for the investigated candidate genes and was provided by Saatzucht Dieckmann.

Oligonucleotide primers amplifying conserved domains of the genes *BvFT1*, *BvFL1* and *BTC1* were designed from genomic sequences with FastPCR [53]. Regions were chosen after analyses of genomic DNA sequence with CODDLE (Codons Optimized to Discover Deleterious Lesions; http://www.proweb.org/coddle/). The primers were pre-screened before labelling in a so called “crash-test” adapted from Weil and Monde [34]. Forward and reverse primers were end dye labelled with Dyomics fluorescent tags DY-681 (700 nm absorption) and DY-781 (800 nm absorption), respectively. PCR amplification was done in a 20 μl volume containing 1 ng pooled DNA, 1 × Taq buffer, 1.5 mM MgCl_2_, 2 mM dNTPs (Invitrogen, Darmstadt, Germany), 0.2 units recombinant TAQ DNA polymerase (Invitrogen, Darmstadt, Germany) and 0.8 pmol primer (biomers.net, Ulm, Germany). The primers were used in a labelled versus non labelled ratio of 3:2 for DY-681 and 4:1 for DY-781 according to Till et al. [38]. PCR was performed on a DNA Engine DYAD thermal cycler (MJ Research Inc., Waltham, MA, USA). PCR steps for the amplification were as follows: an initial denaturation step at 95°C for 5 min followed by 30 cycles of a 30 sec denaturation step at 95°C, 30 sec annealing at 60°C and 60 sec elongation at 72°C. The PCR was concluded with 5 min elongation at 72°C. The crude celery extract (CCE) was extracted as described by Till et al. [38] with a slight modification. We did not dialyze and re-buffer our celery juice as recommended. Instead we used the crude extract for enzymatic mismatch cleavage and tested it against commercial products. The results (data not shown) were identical with those obtained by using Surveyor® endonuclease. For SNP evaluation we only used the 700 nm channel of the LI-COR 4300, because the 800 nm channel did not provide additional information. For heteroduplex formation the PCR product was denatured at 95°C for 10 min and slowly re-annealed by cooling down to 85°C by 2° per sec and further cooling down to 25°C by 0.5°C per sec. The re-annealed PCR product was digested at 42°C for 15 min with crude celery extract (CCE) containing 0.6 μl CCE and 5.4 μl CCE buffer for each 20 μl reaction. The CCE buffer was prepared according to Till et al. [38]. The reaction was stopped with 4 μl 200 mM EDTA. PCR products were cleaned up after endonuclease digestion by Sephadex purification.

Fragment analysis was performed on a LI-COR 4300 DNA analyser using a 6.5% KB ^Plus^ gel matrix (LI-COR®, Bad Homburg, Germany). The gel run was performed at 1,500 V, 40 mA and 40 W for 2 hours and 30 minutes. Acquired data were analysed visually using the software Gelbuddy [54]. For each gel run, an analysis window smaller than the target amplicon size was manually chosen based on image quality and the absence of PCR mispriming artefacts that can occur near the primer binding region [38]. For considering gel bands as digestion fragments, bands in the 700 nm channel were scored and a binary matrix was generated reflecting the presence (1) or absence (0) of all different fragment sizes for each sample.

### Analysis of polymorphisms and haplotypes

For simplification, each unique fragment visible after acrylamide gel electrophoresis was considered as a SNP despite the fact that fragments could also be caused by small indels. SNP densities were calculated as the number of polymorphic SNP loci divided by the total length of screened sequence in kb. Non-reference nucleotide frequencies (NNFs) were calculated for each SNP locus as the number of accessions with the SNP allele deviating from the reference allele of 93161P divided by the number of screened accessions. Average heterozygosity Ht (i.e. gene diversity) for each SNP was calculated with the genetic distance and phylogenetic analysis package DISPAN [55].

Accessions with identical SNP pattern were assigned to the same haplotype. Accessions with no restriction bands on a LI-COR gel were assigned to the reference haplotype H0 (93161P). A haplotype with a frequency of less than 5% was declared rare. Non-reference haplotype frequencies (NHF) were calculated for each haplotype as the number of accessions with a haplotype deviating from the respective reference haplotype (FT1a_H0, FT1b_H0, FL1a_H0, FL1b_H0 or BTC1_H0) divided by the total number of accessions screened per *B. vulgaris* form.

Selected accessions with significant haplotypes associated with bolting rate and survival rate were sequenced via Sanger sequencing with the respective primer combination. To predict the functional impact of the SNPs characteristic of these haplotypes, the web based tools PARSESNP [56] and SIFT [57] were used.

### AFLP analysis and population structure analysis

The population structure of the 268 *B. vulgaris* accessions was analysed with the AFLP (amplified fragment length polymorphism) technique essentially as described by Vos et al. [58]. The following modification was applied: restriction of DNA was carried out with *PstI* instead of *EcoRI*.

Following AFLP marker analysis the population structure was calculated with the software package STRUCTURE version 2.3.4 [33,59]. The optimum number of populations (*k*) was selected after six independent runs with a burn-in of 50,000 iterations followed by 100,000 iterations for each value of *k* (testing from *k* = 1 to *k* = 8). As program parameters for the investigation of the whole panel, the no-admixture model with the correlated allele frequency model was chosen. The most likely value for *k* was determined on the basis of the following criteria: (1) comparison of values for *L(K)* of each *k*; (2) stability of grouping patterns across five runs, and (3) value of ΔK calculated based on the second order rate of change of the likelihood (ΔK = m(|L’(K)|)/s[L(K)]) [60] by the web based interface of STRUCTURE HARVESTER [61].

### Statistical analysis

Association mapping was conducted using the general linear model (GLM) in TASSEL v. 3.0 [62]. An association of a given amplicon with bolting rate, survival rate, or survival rate with bolting rate as cofactor was claimed at an experiment wise alpha level of 0.05 (Bonferroni correction). In case of a significant association of a given amplicon with bolting rate or survival rate, a Dunnett’s *post hoc* test for multiple comparisons of all haplotypes against the reference haplotype H0 was performed. This was performed with the statistical software R [63] separately for each *B. vulgaris* form.

## Competing interests

The authors declare that they have no competing interests.

## Authors’ contributions

SLMF planned, conducted and analysed the EcoTILLING experiments, performed the population genetic analyses and the association test with TASSEL, and drafted and wrote the manuscript. MK planned and conducted the field experiments, carried out the statistical analysis with R and wrote parts of the manuscript concerning phenotypic and statistical data analysis and contributed minor to introduction and discussion. AEM provided sequence information, contributed to data analysis and revised the manuscript. AJS provided the CCE and revised the manuscript. CJ participated in the design of the study and revised the manuscript. FJK conceived the study, and participated in its design, coordination and analysis and helped to draft the manuscript. All authors read and approved the final manuscript.

## Supplementary Material

Additional file 1 **Diagram of mean Log probability for subpopulation calculation.** Mean Log probability *L*(*K*) of results from six parallel calculations for each hypothetic number of subpopulations (*k*) in the range of *k* = 1 to *k* = 8. The *x-axis* shows subpopulations (*k*). The *y*-axis shows the Log probability *L*(*K*). Click here for file

Additional file 2 **Calculation of Delta *K *for subpopulation.** Table output of the Evanno method results. Shown are the number of subpopulations *k*, the mean Log probability and the respective standard deviation (SD), as well as the Delta K (*ΔK*). Click here for file

Additional file 3 **Results of EcoTILLING screens in the three genes *BTC1, BvFL1. *and *BvFL1. ***Listed for each amplicon are the number of successfully screened accessions, the number of detected SNPs, the corresponding SNP density, the number of detected haplotypes, the mean frequency of the reference haplotype H0 from accession 93161P and the range of non-reference haplotype frequency (NHF). SNP densities were calculated as the number of polymorphic SNP loci divided by the total length of screened sequence in kb. NHF were calculated for each haplotype as the number of accessions with haplotype deviating from reference haplotype H0. (DOCX 15 kb) Click here for file

Additional file 4 **Non-reference nucleotide frequencies (NNFs) in three flowering time genes *BvFL1, BvFT1, *and *BTC1 *in divergent *B. vulgaris *forms.** Listed is the NNF for each gene and each *B*. *vulgaris* form as well as the mean NNF for the entire panel. NNFs were calculated as the number of accessions with an allele carrying a SNP (compared to the reference allele in 93161P) divided by the total number of screened accessions. Click here for file

Additional file 5 **Average gene diversity in divergent *B. vulgaris *forms.** Comparison of average gene diversity (Ht) among divergent *B. vulgaris* forms for EcoTILLING amplicons of *BvFL1*, *BvFT1* and *BTC1*. Shown are the gene diversity (Ht) and the standard error (SE) value for each amplicon and across all amplicons within each *B. vulgaris* form as well as across the whole panel. Gene diversity was estimated with the genetic distance and phylogenetic analysis package DISPAN [55]. Click here for file

Additional file 6 **Statistical analysis of haplotypes on bolting rate.** Statistics of haplotypes for the two amplicons of *BvFL1* with significant differences in bolting rate compared with the respective reference haplotypes (FL1a_H0 or FL1b_H0). Shown are the observed haplotypes, their occurrence (n) in each *B*. *vulgaris* form, the average bolting rate, and the corresponding p-value for comparison with the respective reference haplotype. The p-value is Bonferroni corrected to account for the experiment-wise error rate. Click here for file

Additional file 7 **Statistical analysis of haplotypes on survival rate.** Statistics of haplotypes for the two amplicons of *BvFL1* with significant differences in survival rate compared with the respective reference haplotypes (FL1a_H0 or FL1b_H0). Shown are the observed haplotypes, their occurrence (n) in each *B*. *vulgaris* form, the average survival rate, and the corresponding p-value for comparison with the respective reference haplotype. The p-value is Bonferroni corrected to account for the experiment-wise error rate. Click here for file

Additional file 8 **Sequences of the *BvFL1 *haplotypes with impact on survival and bolting rate.** (A) Sequence alignment of reference haplotype FL1a_H0 and FL1a_H6. (B) Multiple sequence alignment of reference haplotype FL1b_H0, FL1b_H3, FL1b_H5, FL1b_H6, and FL1b_H10. Asterisks indicate a single nucleotide polymorphism. Reference nucleotides are marked in yellow and changed nucleotides are marked in red (DOC document viewable with Microsoft Word). Click here for file
