# Supplementary figures and images for: EcoTILLING in Beta vulgaris reveals polymorphisms in the FLC-like gene BvFL1 that are associated with annuality and winter hardiness
Source: BMC Plant Biol. 2013 Mar 25;13:52. doi: 10.1186/1471-2229-13-52 (PMC3636108; doi:10.1186/1471-2229-13-52)

## Slide 1
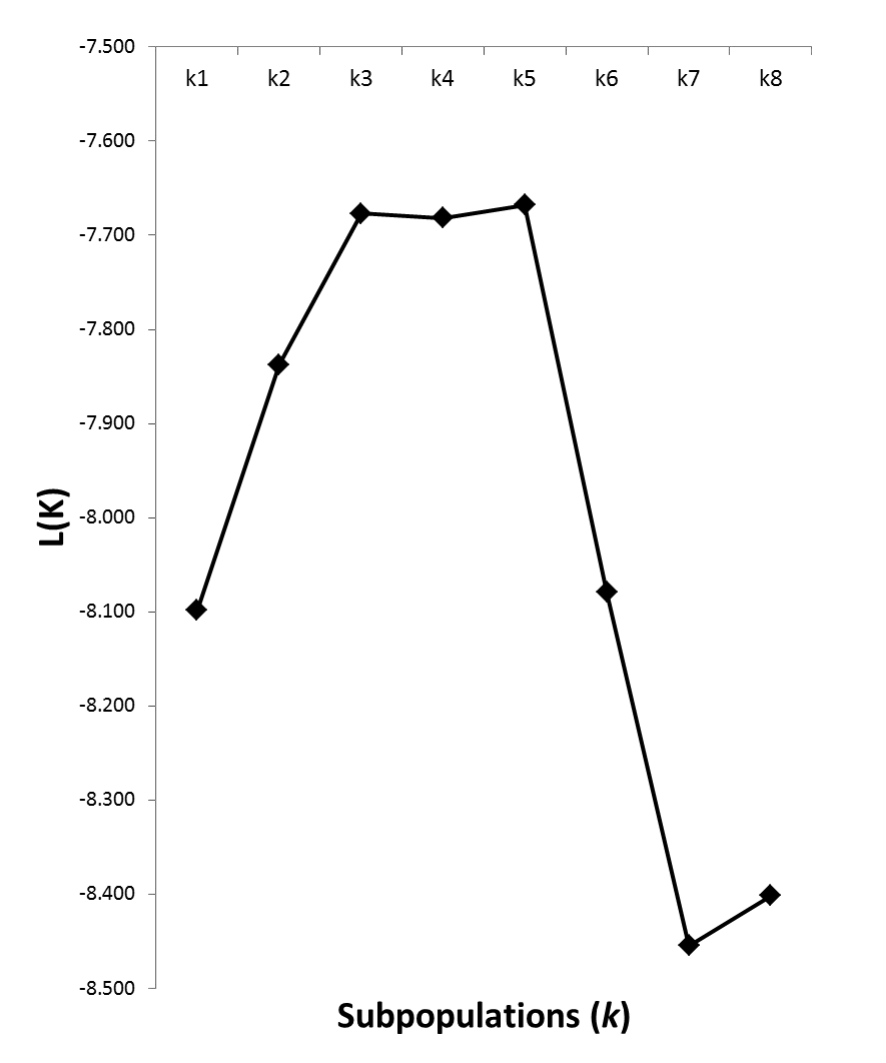

Supplement: Additional file 1 — Diagram of mean Log probability for subpopulation calculation. Mean Log probability L(K) of results from six parallel calculations for each hypothetic number of subpopulations (k) in the range of k = 1 to k = 8. The x-axis shows subpopulations (k). The y-axis shows the Log probability L(K). [file 1471-2229-13-52-S1.ppt]

## Slide 1
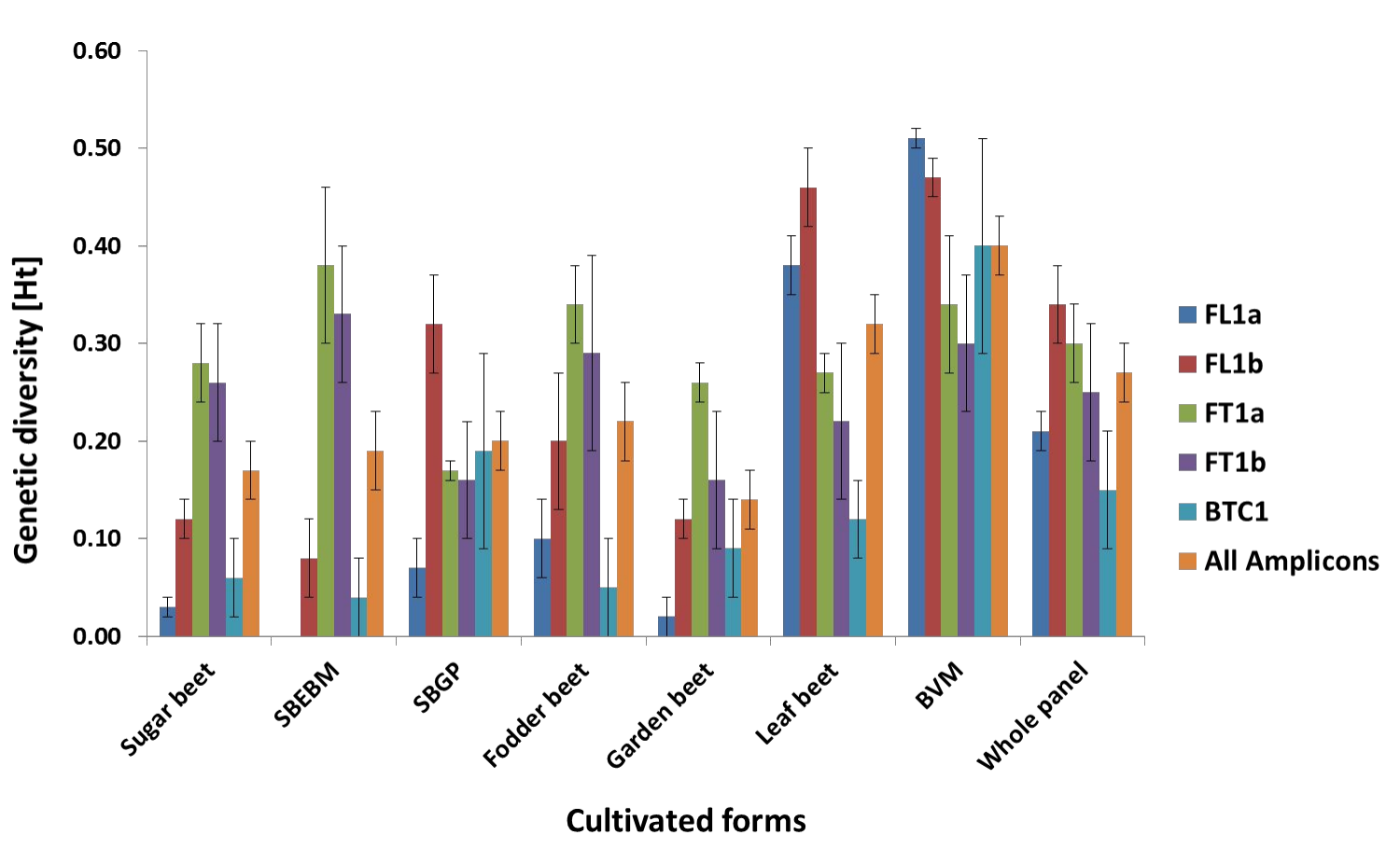

Supplement: Additional file 5 — Average gene diversity in divergent B. vulgaris forms. Comparison of average gene diversity (Ht) among divergent B. vulgaris forms for EcoTILLING amplicons of BvFL1, BvFT1 and BTC1. Shown are the gene diversity (Ht) and the standard error (SE) value for each amplicon and across all amplicons within each B. vulgaris form as well as across the whole panel. Gene diversity was estimated with the genetic distance and phylogenetic analysis package DISPAN [55]. [file 1471-2229-13-52-S5.ppt]
