# Supplementary material for: EcoTILLING in Beta vulgaris reveals polymorphisms in the FLC-like gene BvFL1 that are associated with annuality and winter hardiness
Source: BMC Plant Biol. 2013 Mar 25;13:52. doi: 10.1186/1471-2229-13-52 (PMC3636108; doi:10.1186/1471-2229-13-52)
Supplement: Additional file 2 — Calculation of Delta K for subpopulation. Table output of the Evanno method results. Shown are the number of subpopulations k, the mean Log probability and the respective standard deviation (SD), as well as the Delta K (ΔK). [file 1471-2229-13-52-S2.docx]

### Additional file 2 – Calculation of Delta *K* for subpopulation

Table output of the Evanno method results. Shown are the number of subpopulations *k*, the mean Log probability and the respective standard deviation (SD), as well as the Delta *K* (Δ*K*).

| **# *k*** | **Mean *L*(*K*)** | **SD *L*(*K*)** | **Δ*K*** |
| --- | --- | --- | --- |
| 1 | -8098.50 | 0.81 | NA |
| 2 | -7837.60 | 4.61 | 21.76 |
| 3 | -7676.98 | 2.76 | 60.04 |
| 4 | -7681.92 | 21.98 | 0.85 |
| 5 | -7668.15 | 28.68 | 14.82 |
| 6 | -8079.55 | 105.92 | 0.35 |
| 7 | -8454.38 | 51.58 | 8.30 |
| 8 | -8401.23 | 82.29 | 0.25 |
