# Supplementary material for: EcoTILLING in Beta vulgaris reveals polymorphisms in the FLC-like gene BvFL1 that are associated with annuality and winter hardiness
Source: BMC Plant Biol. 2013 Mar 25;13:52. doi: 10.1186/1471-2229-13-52 (PMC3636108; doi:10.1186/1471-2229-13-52)
Supplement: Additional file 3 — Results of EcoTILLING screens in the three genes BTC1, BvFL1. and BvFL1. Listed for each amplicon are the number of successfully screened accessions, the number of detected SNPs, the corresponding SNP density, the number of detected haplotypes, the mean frequency of the reference haplotype H0 from accession 93161P and the range of non-reference haplotype frequency (NHF). SNP densities were calculated as the number of polymorphic SNP loci divided by the total length of screened sequence in kb. NHF were calculated for each haplotype as the number of accessions with haplotype deviating from reference haplotype H0. (DOCX 15 kb) [file 1471-2229-13-52-S3.docx]

### Additional File 3 - Results of EcoTILLING screens in the three genes *BvBTC1*, *BvFL1*, and *BvFL1*

Listed for each amplicon are the number of successfully screened accessions, the number of detected SNPs, the corresponding SNP density, the number of detected haplotypes, the mean frequency of the reference haplotype H0 from accession 93161P and the range of non-reference haplotype frequency (NHF). SNP densities were calculated as the number of polymorphic SNP loci divided by the total length of screened sequence in kb. NHF were calculated for each haplotype as the number of accessions with haplotype deviating from reference haplotype H0.

| **Amplicon** | **screened accessions** | **Detected SNPs** | **SNP density** | **Haplotypes detected per amplicon** | **Mean frequency of H0** | **Range of NHF** |
| --- | --- | --- | --- | --- | --- | --- |
| FL1a | 219 | 3 | 3.28 | 7 | 0.77 | 0.03 – 0.38 |
| FL1b | 239 | 7 | 9.82 | 18 | 0.57 | 0.01 - 0.32 |
| FT1a | 242 | 4 | 4.09 | 12 | 0.55 | 0.01 – 0.20 |
| FT1b | 248 | 5 | 7.91 | 14 | 0.49 | 0.01 – 0.33 |
| BTC1 | 237 | 2 | 2.01 | 4 | 0.87 | 0.01 – 0.28 |
| Over all amplicons |  | 21 | 5.3* | 55 | 0.65 |  |

* mean SNP density over all amplicons
