# Supplementary material for: EcoTILLING in Beta vulgaris reveals polymorphisms in the FLC-like gene BvFL1 that are associated with annuality and winter hardiness
Source: BMC Plant Biol. 2013 Mar 25;13:52. doi: 10.1186/1471-2229-13-52 (PMC3636108; doi:10.1186/1471-2229-13-52)
Supplement: Additional file 4 — Non-reference nucleotide frequencies (NNFs) in three flowering time genes BvFL1, BvFT1, and BTC1 in divergent B. vulgaris forms. Listed is the NNF for each gene and each B. vulgaris form as well as the mean NNF for the entire panel. NNFs were calculated as the number of accessions with an allele carrying a SNP (compared to the reference allele in 93161P) divided by the total number of screened accessions. [file 1471-2229-13-52-S4.docx]

### Additional File 4 - Non-reference nucleotide frequencies in flowering time genes in divergent *B*. *vulgaris* forms

Non-reference nucleotide frequencies (NNFs) in three flowering time genes *BvFL1*, *BvFT1*, and *BvBTC1* in divergent *B*. *vulgaris* forms. Listed is the NNF for each gene and each *B*. *vulgaris* form as well as the mean NNF for the entire panel. NNFs were calculated as the number of accessions with an allele carrying a SNP (compared to the reference allele in 93161P) divided by the total number of screened accessions.

| **Gene** | **NNF Sugar beet** | **NNF Fodder beet** | **NNF Garden beet** | **NNF Leaf beet** | **NNF BVM^a)^** | **Mean NNF** |
| --- | --- | --- | --- | --- | --- | --- |
| *BvFL1* | 0.05 | 0.11 | 0.04 | 0.36 | 0.55 | 0.18 |
| *BvFT1* | 0.14 | 0.21 | 0.12 | 0.15 | 0.23 | 0.17 |
| *BvBTC1* | 0.05 | 0.03 | 0.04 | 0.06 | 0.3 | 0.07 |

a) BVM = *B.* *vulgaris* ssp. *maritima*
