# Supplementary material for: EcoTILLING in Beta vulgaris reveals polymorphisms in the FLC-like gene BvFL1 that are associated with annuality and winter hardiness
Source: BMC Plant Biol. 2013 Mar 25;13:52. doi: 10.1186/1471-2229-13-52 (PMC3636108; doi:10.1186/1471-2229-13-52)
Supplement: Additional file 6 — Statistical analysis of haplotypes on bolting rate. Statistics of haplotypes for the two amplicons of BvFL1 with significant differences in bolting rate compared with the respective reference haplotypes (FL1a_H0 or FL1b_H0). Shown are the observed haplotypes, their occurrence (n) in each B. vulgaris form, the average bolting rate, and the corresponding p-value for comparison with the respective reference haplotype. The p-value is Bonferroni corrected to account for the experiment-wise error rate. [file 1471-2229-13-52-S6.docx]

### Additional file 6 – Statistical analysis of haplotypes on bolting rate

Statistics of haplotypes for the two amplicons of *BvFL1* with significant differences in bolting rate compared with the respective reference haplotypes (FL1a_H0 or FL1b_H0). Shown are the observed haplotypes, their occurrence (n) in each *B*. *vulgaris* form, the average bolting rate, and the corresponding p-value for comparison with the respective reference haplotype. The p-value is Bonferroni corrected to account for the experiment-wise error rate.

| **haplotype** |  | **Sugar beet** | **Fodder beet** | **Garden beet** | **Leaf beet** | **BVM^b)^** |
| --- | --- | --- | --- | --- | --- | --- |
| All | average BR before winter | 0% | 1% | 0% | 4% | 8% |
| FL1a_H0 | n^a)^ | 66 | 32 | 42 | 20 | 7 |
|  | average BR before winter | 0% | 1% | 1% | 5% | 1% |
| FL1a_H6 | n | - | 4 | - | 5 | 3 |
|  | average BR before winter | - | 0% | - | 5% | 55% |
|  | p-Value | - | 0.93 | - | 1 | <1e-06 |
| FL1b_H0 | n | 64 | 16 | 40 | 12 | 5 |
|  | average BR before winter | 0% | 1% | 0% | 8% | 2% |
| FL1b_H5 | n | - | - | - | - | 1 |
|  | average BR before winter | - | - | - | - | 75% |
|  | p-Value | - | - | - | - | <1e-04 |
| FL1b_H6 | n | - | - | 2 | 4 | 2 |
|  | average BR before winter | - | - | 6% | 0% | 0% |
|  | p-Value | - | - | <1e-09 | 0.461 | 1 |
| FL1b_H10 | n | - | 1 | 1 | 2 | 2 |
|  | average BR before winter | - | 0% | 0% | 0% | 11% |
|  | p-Value | - | 1 | 1 | 0.434 | 0.0356 |

a) n: Number of accessions carrying the given haplotype

b) BVM = *Beta* *vulgaris* ssp. *Maritima*

c) BR = bolting rate
