# Supplementary material for: EcoTILLING in Beta vulgaris reveals polymorphisms in the FLC-like gene BvFL1 that are associated with annuality and winter hardiness
Source: BMC Plant Biol. 2013 Mar 25;13:52. doi: 10.1186/1471-2229-13-52 (PMC3636108; doi:10.1186/1471-2229-13-52)
Supplement: Additional file 7 — Statistical analysis of haplotypes on survival rate. Statistics of haplotypes for the two amplicons of BvFL1 with significant differences in survival rate compared with the respective reference haplotypes (FL1a_H0 or FL1b_H0). Shown are the observed haplotypes, their occurrence (n) in each B. vulgaris form, the average survival rate, and the corresponding p-value for comparison with the respective reference haplotype. The p-value is Bonferroni corrected to account for the experiment-wise error rate. [file 1471-2229-13-52-S7.docx]

### Additional file 7 – Statistical analysis of haplotypes on survival rate

Statistics of haplotypes for the two amplicons of *BvFL1* with significant differences in survival rate compared with the respective reference haplotypes (FL1a_H0 or FL1b_H0). Shown are the observed haplotypes, their occurrence (n) in each *B*. *vulgaris* form, the average survival rate, and the corresponding p-value for comparison with the respective reference haplotype. The p-value is Bonferroni corrected to account for the experiment-wise error rate.

|  |  | **Sugar beet** | **Fodder beet** | **Garden beet** | **Leaf beet** | **BVM^b)^** |
| --- | --- | --- | --- | --- | --- | --- |
| All | average SR | 40% | 23% | 20% | 29% | 40% |
| FL1a_H0 | n^a)^ | 66 | 32 | 42 | 20 | 7 |
|  | average SR | 39% | 23% | 21% | 22% | 39% |
| FL1a_H6 | n | - | 4 | - | 5 | 3 |
|  | average SR | - | 21% | - | 35% | 13% |
|  | p-Value | - | 1 | - | 0.339 | 0.0143 |
| FL1b_H0 | n | 64 | 16 | 40 | 12 | 5 |
|  | average SR | 39% | 27% | 20% | 19% | 36% |
| FL1b_H3 | n | 1 | - | - | 9 | 4 |
|  | average SR | 40% | - | - | 37% | 43% |
|  | p-Value | 1 | - | - | 0.0116 | 1 |

a) n: Number of accessions carrying the given haplotype

b) BVM = *Beta* *vulgaris* ssp. *maritima*

c) SR = survival rate
