# Supplementary material for: EcoTILLING in Beta vulgaris reveals polymorphisms in the FLC-like gene BvFL1 that are associated with annuality and winter hardiness
Source: BMC Plant Biol. 2013 Mar 25;13:52. doi: 10.1186/1471-2229-13-52 (PMC3636108; doi:10.1186/1471-2229-13-52)
Supplement: Additional file 8 — Sequences of the BvFL1 haplotypes with impact on survival and bolting rate. (A) Sequence alignment of reference haplotype FL1a_H0 and FL1a_H6. (B) Multiple sequence alignment of reference haplotype FL1b_H0, FL1b_H3, FL1b_H5, FL1b_H6, and FL1b_H10. Asterisks indicate a single nucleotide polymorphism. Reference nucleotides are marked in yellow and changed nucleotides are marked in red (DOC document viewable with Microsoft Word). [file 1471-2229-13-52-S8.docx]

**(A)**

FL1a_H0 TCGGACTTTCCCTATAAGCTTAAGAAAATAAATGAATGATACTATAAATAGATAAAAATA

FL1a_H6 TCGGACTTTCCCTATAAGCTTAAGAAAATAAATGAATGATACTATAAATAGATAAAAATA

FL1a_H0 GCCTAAATAGGGCATCCACAGCTAGTAAGGAGATAAACTGATAAACTAAAAGTCTAAAAC

FL1a_H6 GCCTAAATAGGGCATCCACAGCTAGTAAGGAGATAAACTGATAAACTAAAAGTCTAAAAC

FL1a_H0 TAAAACGAAAACGATAAGTTGCACTAAAATCTAATACTTATCTAACATTTCCACGTGTAA

FL1a_H6 TAAAACGAAAACGATAAGTTGCACTAAAATCTAATACTTATCTAACATTTCCACGTGTAA

FL1a_H0 TAAATAAAACATGGGGCCCAATGATTAATCTTCAACTGACCTCTCCCTCCAATCGCGTAT

FL1a_H6 TAAATAAAACATGGGGCCCAATGATTAATCTTCAACTGACCTCTCCCTCCAATCGCGTAT

FL1a_H0 TCTAAAAATCATTTCACTATCTCTTTCATCTTTTTTTTTCCGGCATTTTTTTTTCATTTC

FL1a_H6 TCTAAAAATCATTTCACTATCTCTTTCATCTTTTTTTTTCCGGCATTTTTTTTTCATTTC

FL1a_H0 CTCTCTCTCTTACTCTATACTACTCTAGGGTCTATATGATACTAGTATTCAGTAGTAGCA

FL1a_H6 CTCTCTCTCTTACTCTATACTACTCTAGGGTCTATATGATACTAGTATTCAGTAGTAGCA

FL1a_H0 GTAATAGAAGTACTATCAGTTTTCTCTTCCTTTTGAAATAAAATTAGTATTCTATTCAAT

FL1a_H6 GTAATAGAAGTACTATCAGTTTTCTCTTCCTTTTGAAATAAAATTAGTATTCTATTCAAT

FL1a_H0 TAAAATCGATCCACTAGTCTAAATACTAGTACTATTATCAGAGGAGAAGAAAGGACAGAG

FL1a_H6 TAAAATCGATCCACTAGTCTAAATACTAGTACTATTATCAGAGGAGAAGAAAGGACAGAG

FL1a_H0 AGAGTGAGAGAAATTGCAGCGACGAAGACAGAGAAAGGTATTTGGATAAGGATGGGAAGA

FL1a_H6 AGAGTGAGAGAAATTGCAGCGACGAAGACAGAGAAAGGTATTTGGATAAGGATGGGAAGA

FL1a_H0 AGGAAGATAGAGATGAAAAGAATTGAAGATAAAAGTAGTCGTCARGTTACATTTTCAAAG

FL1a_H6 AGGAAGATAGAGATGAAAAGAATTGAAGATAAAAGTAGTCGTCARGTTACATTTTCAAAG

FL1a_H0 CGGCGTTCTGGTCTTATCAAAAAAGCTCGCGAACTCTCTATCCTTTGTGATGTCGATGTT

FL1a_H6 CGGCGTTCTGGTCTTATCAAAAAAGCTCGCGAACTCTCTATCCTTTGTGATGTCGATGTT

FL1a_H0 GCTGTTCTTGTTTTCTCTAATCGTGGCCGTCTTTACGAATTCGTCAATAGTTCTTCTTCT

FL1a_H6 GCTGTTCTTGTTTTCTCTAATCGTGGCCGTCTTTACGAATTCGTCAATAGTTCTTCTTCT

FL1a_H0 TCCAGGTTTTTCTTCATCTCTTTCCTTGTTATTAGTTTTTTCTTTTTCAATTTTCAAGTT

FL1a_H6 TCCAGGTTTTTCTTCATCTCTTTCCTTGTTATTAGTTTTTTCTTTTTCAATTTTCAAGTT

FL1a_H0 TAATTTTAATGGTATGCTTTGTTGAATAGTTTCGGTTGTTAATGGCGGAATTTTGTTGTT

FL1a_H6 TAATTTTAATGGTATGCTTTGTTGAATAGTTTCGGTGGTTAATGGCGGAATTTTGTTGTT

*

FL1a_H0 TAGTTTTTTCGGTGTGTTTTGTTGTTTTGTGATTCTAGGTTTTAGATGATTTTGCTTGAT

FL1a_H6 TAGTTTTTTCGGTGTGTTTTGTTGTTTTGTGATTCTAGGTTTTAGATGATTTTGCTTGAT

FL1a_H0 TTCATGAATTTTGAATTTTGGAGGTTTTTGGTTTTACCGTATAAAATTGAATGATTTTGT

FL1a_H6 TTCATGAATTTTGAATTTTGGAGGTTTTTGGTTTTACCGTATAAAATTGAATGATTTTGT

FL1a_H0 CTGTAACGATTCACGTG

FL1a_H6 CTGTAACGATTCACGTG

**(B)**

FL1b_H0 GCTGATAGTCTGTCCCTTTTGTCAATCAGAGAGTTCTTTGTCTCCAATGTCATTCTTATT

FL1b_H03 GCTGATAGTCTGTCCCTTTTGTCAATCAGAGAGTTCTTTGTCTCCAATGTCATTCTTATT

FL1b_H05 GCTGATAGTCTGTCCCTTTTGTCAATCAGAGAGTTCTTTGTCTCCAATGTCATTCTTATT

FL1b_H06 GCTGATAGTCTGTCCCTTTTGTCAATCAGAGAGTTCTTTGTCTCCAATGTCATTCTTATT

FL1b_H10 GCTGATAGTCTGTCCCTTTTGTCAATCAGAGAGTTCTTTGTCTCCAATGTCATTCTTATT

FL1b_H0 CAAGGCCATGAATAAATTTTCCTACTTTGAATATTCATAATTTCATATCCTTCATGCTTC

FL1b_H03 CAAGGCCATGAATAAATTTTCCTACTTTGAATATTCATAATTTCATATCCTTCATGCTTC

FL1b_H05 CAAGGCCATGAATAAATTTTCCTACTTTGAATATTCATAATTTCATATCCTTCATGCTTC

FL1b_H06 CAAGGCCATGAATAAATTTTCCTACTTTGAATATTCATAATTTCATATCCTTCATGCTTC

FL1b_H10 CAAGGCCATGAATAAATTTTCCTACTTTGAATATTCATAATTTCATATCCTTCATGCTTC

FL1b_H0 TGTCCGTACAACTTTTCGTTCTATTCTCTGACAATTCTGGAGTTCTTTTTGCTTTTGATA

FL1b_H03 TGTCCGTACAACTTTTCGTTCTATTCTCTGACAATTCTGGAGTTCTTTTTGCTTTTGATA

FL1b_H05 TGTCCGTACAACTTTTCGTTCTATTCTCTGACAATTCTGGAGTTCTTTTTGCTTTTGATA

FL1b_H06 TGTCCGTACAACTTTTCGTTCTATTCTCTGACAATTCTGGAGTTCTTTTTGCTTTTGATA

FL1b_H10 TGTCCGTACAACTTTTCGTTCTATTCTCTGACAATTCTGGAGTTCTTTTTGCTTTTGATA

FL1b_H0 GCAGAGTTCACCTTCTAGTTGTGCAGAAGTTCAAACATGTGGTGAGCTAGTAAAATCAGT

FL1b_H03 GCAGAGTTCACCTTCTAGTTGTGCAGAAGTCCAAACATGTGGTGAGCTAGTAAAATCAGT

FL1b_H05 GCAGAGTTCACCTTCTAGTTGTGCAGAAGTCCAAACATGTGGTGAGCTAGTAAAATCAGT

FL1b_H06 GCAGAGTTCACCTTCTAGTTGTGCAGAAGTCCAAACATGTGGTGAGCTAGTAAAATCAGT

FL1b_H10 GCAGAGTTCACCTTCTAGTTGTGCAGAAGTTCAAACATGTGGTGAGCTAGTAAAATCAGT

*

FL1b_H0 TGAAGGGCAAGTACTCAATATTCTATTTACTTCTGACGATGACTTCTCCATGTTCCATAA

FL1b_H03 TGAAGGGCAAGTACTCAATTTTCTATTTACTTCTGACGATGACTTCTCCATGTTCCATAA

FL1b_H05 TGAAGGGCAAGTACTCAATTTTCTATTTACTTCTGACGATGACTTCTCCATGTTCCATAA

FL1b_H06 TGAAGGGCAAGTACTCAATTTTCTATTTACTTCTGACGATGACTTCTCCATGTTCCATAA

FL1b_H10 TGAAGGGCAAGTACTCAATTTTCTATTTACTTCTGACGATGACTTCTCCATGTTCCATAA

*

FL1b_H0 TTATGGTCAACTTACAGGTACCTAGAAGGACCAGAGCTTGAAAATCTTAGGCTTGAGGAC

FL1b_H03 TTATGGTCAACTTACAGGTACCTAGAAGGACCAGAGCTTGAAAATCTTAGGCTTGAGGAC

FL1b_H05 TTATGGTCAACTTACAGGTACCTAGAAGGACCAGAGCTTGAAAATCTTAGGCTTGAGGAC

FL1b_H06 TTATGGTCAACTTACAGGTACCTAGAAGGACCAGAGCTTGAAAATCTTAGGCTTGAGGAC

FL1b_H10 TTATGGTCAACTTACAGGTACCTAGAAGGACCAGAGCTTGAAAATCTTAGGCTTGAGGAC

FL1b_H0 TTCATGAGGCTGGAGAGGCAACTAGCTGATGCCCTTATACAGACCAGAACCCGAAAGGTT

FL1b_H03 TTCATGAGGCTGGAGAGGCAACTAGCTGATGCCCTTGTACAGACCAGAACCCGAAAGGTT

FL1b_H05 TTCATGAGGCTGGAGAGGCAACTAGCTGATGCCCTTATACAGACCAGAACCCGAAAGGTT

FL1b_H06 TTCATGAGGCTGGAGAGGCAACTAGCTGATGCCCTTATACAGACCAGAACCCGAAAGGTT

FL1b_H10 TTCATGAGGCTGGAGAGGCAACTAGCTGATGCCCTTGTACAGACCAGAACCCGAAAGGTT

*

FL1b_H0 CTCTTCTTCCCTTGTAAACCATTTAGTTTGATGATCTACCACGTGGTTGGGTTGGAAGCT

FL1b_H03 CTCTTCTTCCCTTGTAAACCATTTAGTTTGATGATCTACCACGTGGTTGGGTTGGAAGCT

FL1b_H05 CTCTTCTTCCCTTGTAATCCATTTAGTTTGATGATCTACCACGTGGTTGGGTTGGAAGCT

FL1b_H06 CTCTTCTTCCCTTGTAATCCATTTAGTTTGATGATCTACCACGTGGTTGGGTTGGAAGCT

FL1b_H10 CTCTTCTTCCCTTGTAATCCATTTAGTTTGATGATCTACCACGTGGTTGGGTTGGAAGCT

*

FL1b_H0 GGATAGTTTATATGTCTTTACCCTTTCTCTATGCTTACACTTTGCATATTTGATGGATGG

FL1b_H03 GGATAGTTTATATTTCTTTACCCTTTTTCTACGCTTACACTTTGCATATTTGATGGATGG

FL1b_H05 GGATAGTTTATATTTCTTTACCCTTTCTCTACGCTTACACTTTGCATATTTGATGGATGG

FL1b_H06 GGATAGTTTATATGTCTTTACCCTTTCTCTACGCTTACACTTTGCATATTTGATGGATGG

FL1b_H10 GGATAGTTTATATTTCTTTACCCTTTTTCTACGCTTACACTTTGCATATTTGATGGATGG

* * *

FL1b_H0 AGGTTCACTAATTTAAATTTGCAAGATCAAAGAGCTACTGTTCTCTGAAGCAAAGATGCG

FL1b_H03 AGGTTCACTAATTTAAATTTGCAAGATCAAAGAGCTACTGTTCTCTGAAGCAAAGATGCG

FL1b_H05 AGGTTCACTAATTTAAATTTGCAAGATCAAAGAGCTACTGTTCTCTGAAGCAAAGATGCG

FL1b_H06 AGGTTCACTAATTTAAATTTGCAAGATCAAAGAGCTACTGTTCTCTGAAGCAAAGATGCG

FL1b_H10 AGGTTCACTAATTTAAATTTGCAAGATCAAAGAGCTACTGTTCTCTGAAGCAAAGATGCG

FL1b_H0 ACTGTATTTTATGCATCGTGGTGTTGGAGTCA

FL1b_H03 ACTGTATTTTATGCATCGTGGTGTTGGAGTCA

FL1b_H05 ACTGTATTTTATGCATCGTGGTGTTGGAGTCA

FL1b_H06 ACTGTATTTTATGCATCGTGGTGTTGGAGTCA

FL1b_H10 ACTGTATTTTATGCATCGTGGTGTTGGAGTCA

### Additional file 8 - Sequences of the *BvFL1* haplotypes with impact on survival and bolting rate

(A) Sequence alignment of reference haplotype FL1a_H0 and FL1b_H6. (B) Multiple sequence alignment of reference haplotype FL1b_H0, FL1b_H3, FL1b_H5, FL1b_H6, and FL1b_H10. Asterisks indicate a single nucleotide polymorphism. Reference nucleotides are marked in yellow and changed nucleotides are marked in red (DOC document viewable with Microsoft Word).
